# Low-Power Long-Term Ambulatory Electrocardiography Monitor of Three Leads with Beat-to-Beat Heart Rate Measurement in Real Time

**DOI:** 10.3390/s23198303

**Published:** 2023-10-08

**Authors:** Frank Martínez-Suárez, José Alberto García-Limón, Jorge Enrique Baños-Bautista, Carlos Alvarado-Serrano, Oscar Casas

**Affiliations:** 1 Bioelectronics Section, Department of Electrical Engineering, Centro de Investigación y de Estudios Avanzados del Instituto Politécnico Nacional (Cinvestav), Mexico City 07360, Mexico; josea.garcial@cinvestav.mx (J.A.G.-L.); jorge.banosb@cinvestav.mx (J.E.B.-B.); 2 Instrumentation, Sensors and Interfaces Group, Universitat Politècnica de Catalunya (Barcelona Tech), 08860 Barcelona, Spain; jaime.oscar.casas@upc.edu

**Keywords:** ambulatory ECG monitor, heart rate, real-time QRS detection, ESP32, ADS1294, long-term ECG recordings

## Abstract

A low-power long-term ambulatory ECG monitor was developed for the acquisition, storage and processing of three simultaneous leads DI, aVF and V2 with a beat-to-beat heart rate measurement in real time. It provides long-term continuous ECG recordings until 84 h. The monitor uses a QRS complex detection algorithm based on the continuous wavelet transform with splines, which automatically selects the scale for the analysis of ECG records with different sampling frequencies. It includes a lead-off detection to continuously monitor the electrode connections and a real-time system of visual and acoustic alarms to alert users of abnormal conditions in its operation. The monitor presented is based in an ADS1294 analogue front end with four channels, 24-bit analog-to-digital converters and programmable gain amplifiers, a low-power dual-core ESP32 microcontroller, a microSD memory for data storage in a range of 4 GB to 32 GB and a 1.4 in thin-film transistor liquid crystal display (LCD) variant with a resolution of 128 × 128 pixels. It has programmable sampling rates of 250, 500 and 1000 Hz; a bandwidth of 0 Hz to 50% of the selected sampling rate; a CMRR of −105 dB; an input margin of ±2.4 V; a resolution of 286 nV; and a current consumption of 50 mA for an average battery life of 84 h. The ambulatory ECG monitor was evaluated with the commercial data-acquisition system BIOPAC MP36 and its module for ECG LABEL SS2LB, simultaneously comparing the morphologies of two ECG records and obtaining a correlation of 91.78%. For the QRS detection in real time, the implemented algorithm had an error less than 5%. The developed ambulatory ECG monitor can be used for the analysis of the dynamics of the heart rate variability in long-term ECG records and for the development of one’s own databases of ECG recordings of normal subjects and patients with cardiovascular and noncardiovascular diseases.

## 1. Introduction

Cardiovascular diseases are the leading cause of death worldwide [1]. Therefore, there is a need for ambulatory (Holter) electrocardiography (ECG) monitors that can supplement traditional medical and clinical care, allowing for the noninvasive evaluation of electrocardiographic abnormalities over long periods of time and permitting the patients to conduct their normal daily activities. In contrast to the standard 12-lead ECG recordings of brief duration used in clinical examinations, where these intermittent cardiac abnormalities during the day may be undetected, the Holter ECG monitor allows one to continuously examine a patient over an extended time of 24 to 48 h, providing a long-term continuous recording [2]. 

The main uses of long-term ECG recordings in clinical practice are in the diagnosis and assessment of cardiac symptoms, the prognosis assessment and risk stratification of cardiac disease populations and the evaluation of therapeutic interventions [2]. Two parameters with great clinical value as risk predictors of cardiovascular death and arrhythmic events that can be measured with these recordings are the RR interval changes between consecutive beats and after spontaneous ventricular premature complexes, which can be used for the analysis of the heart rate variability (HRV) and the heart rate turbulence (HRT), respectively [3].

The evolution of technology in microelectronic circuits has led Holter devices to have a decrease in weight, size and power consumption and a high-performance computing capacity, allowing for the implementation of algorithms to provide an online analysis by using more complex signal-processing methods [4]. Currently, the long-term continuous ambulatory electrocardiography (CAECG) monitors record ECG data that permit the analysis of dynamic and transient electrocardiographic changes for up 24 to 48 h or more [2,5,6]. Some devices can record and store from 3 to 12 simultaneous leads for 24 h with sampling frequencies of up to 4096 Hz and perform continuous beat-by-beat monitoring online for the real-time measurement of electrocardiographic parameters [7,8,9].

In recent years, we proposed two prototypes of CAECG monitors based on the Field Programmable Gate Array (FPGA) with the beat-to-beat real-time detection of the QRS complex [10] and the QRS complex and T-wave end [11]. In [12], we presented a CAECG monitor based on the low-power dual-core ESP32 microcontroller with three selectable sampling rates, simultaneous acquisition and the storage of three leads, a beat-to-beat heart rate measurement in real time and lead-off detection for continuous periods until 84 h.

The developed CAECG monitor is a low-power and low-cost hardware system that allows for a very simple adaptation to the change in leads, modifying the electrodes’ position. It also allows one to increase the number of ECG leads or the measurement of different biopotentials by replacing the ADS1294 (four-channel, analog-to-digital converter with an integrated ECG front end) with ADS1296, ADS1298 or ADS1299 by making small adjustments to the software and hardware. The detection algorithms of the ECG characteristic points used in [10,11,12] were based on the continuous wavelet transform (CWT) with splines [13]. This technique allows for the evaluation of the CWT on any integer scale, reducing noise, interference and artifacts more efficiently [14].

In this work, we present an extended version of the CAECG monitor presented in [12], including the description of the components of the communication and charging circuit, the real-time alarm system, the real-time detection algorithm of the QRS complex based in the CWT with splines and the ECG record of three leads with the QRS complex detection from a test subject acquired by the CAECG monitor.

## 2. Materials and Methods

**Figure 1:**
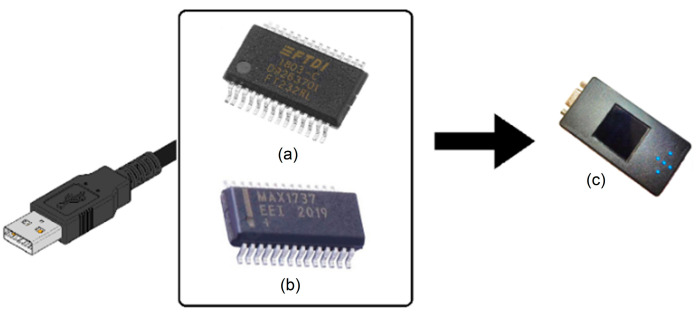
Main components of the charging circuit and USB communication with the CAECG monitor. (**a**) FT232R. (**b**) MAX1736. (**c**) CAECG monitor.

The proposed solution is composed of two elements: the first is the charging and communication circuit that has the FT232R [15] as its nucleus for communication with the ESP32 and the MAX1736 [16] to control the battery charge of the CAECG monitor (Figure 1).

**Figure 2:**
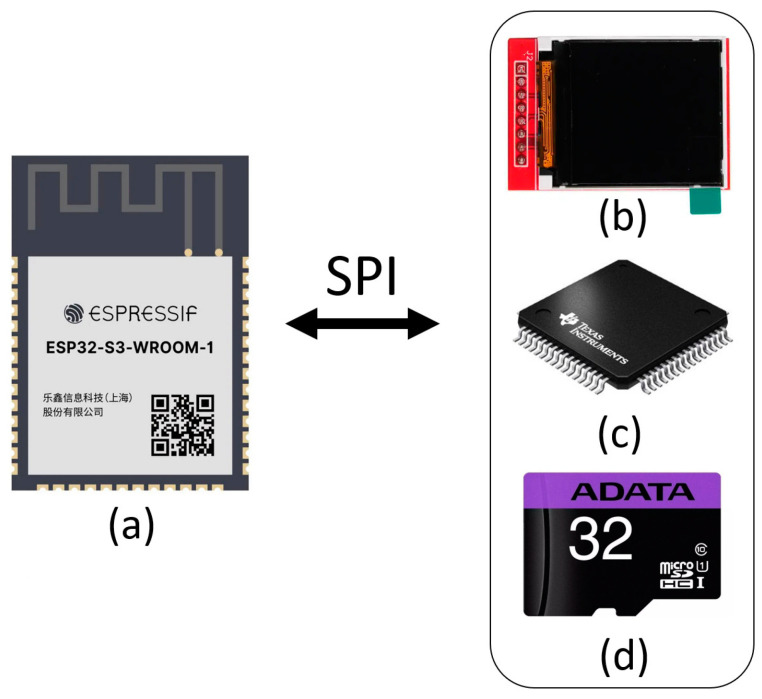
Main components of the CAECG monitor of 3 leads with R-wave detection in real time, communicated by SPI protocol. (**a**) ESP32. (**b**) LCD screen. (**c**) ADS1294. (**d**) MicroSD memory.

The second element is the CAECG monitor, which has at its core an ADS1294 that is responsible for signal digitalization, an LCD screen as a user interface, a microSD memory for information storage and the ESP32 microcontroller to manage the interaction between the above components (Figure 2). The firmware was developed in Arduino version 1.8.2, for which it was necessary to add the libraries for the ESP32, the microSD memory and the LCD screen.

### 2.1. Main Components of the Communication and Charging Circuit

The charging circuit has two fundamental elements: the FT232R, which allows for the communication between the CAECG monitor and a personal computer for firmware updates and information exchange, and the MAX1736 to control battery charging.

The FT232R is a single-chip USB-to-UART interface with an asynchronous serial-data-transfer interface; all the USB protocols are handled on a chip, and no USB-specific firmware programming is required [15].

The MAX1736 is a simple, low-cost, single-cell lithium-ion (Li+) battery charger for small portable applications. The MAX1736EUT42 is preset to a battery-regulation voltage of 4.2 V and initiates charging in one of four ways [16]:

- By inserting the battery.
- By powering up the charger.
- By meeting the battery voltage threshold.
- By external manipulation of the EN pin.

#### Communication and Charging Module

**Figure 3:**
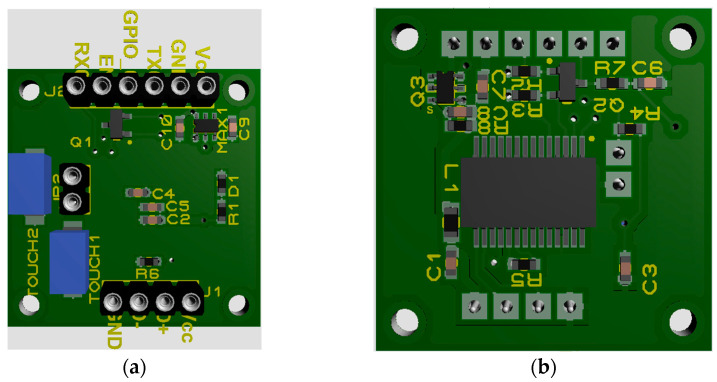
Printed circuit board. (**a**) Upper side. (**b**) Lower side.

For the design of the communication and charging module, the ESP32 DEVKIT V1 development board was taken as a reference, and the charging circuit was added. The printed circuit board was designed with Proteus 8.11 software on two sides: on the upper side there is the MAX1736, and on the lower side is the FT232R, as shown in Figure 3.

**Figure 4:**
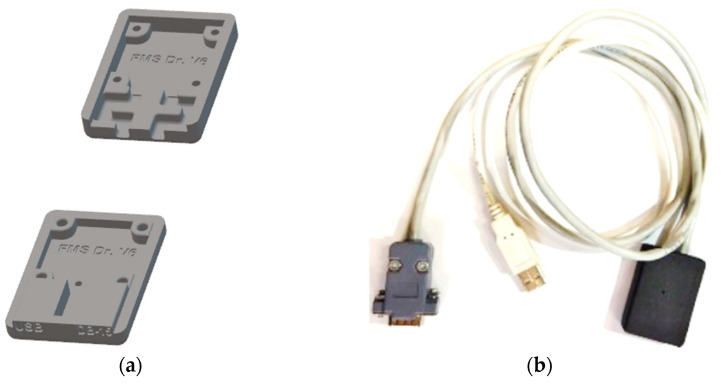
Communication and charging module: (**a**) 3D box model. (**b**) Final module.

This circuit has pins at one extreme to connect a USB cable to communicate with a PC (Figure 3, connector J1), and at the other extreme, the pins are connected to a DB-15 connector to charge the battery and communicate with the ESP32 of the CAECG monitor (Figure 3, connector J2). To protect the circuit, a 3D box was designed with Autodesk Inventor 2022, and the design was printed with a 3D printer with PLA (Figure 4).

### 2.2. Fundamental Components of the CAECG Monitor

The CAECG monitor developed has four main elements that are each responsible for managing a different function:

- Storage (microSD memory).
- Digitization of the ECG signal (ADS1294).
- User interaction with the prototype (LCD screen and touch buttons).
- Device management (ESP32).

The ADS1294 from Texas Instruments is a delta–sigma (ΔΣ) analog-to-digital converter with simultaneous sampling from 1 to 4 channels with a configurable gain factor from 1 to 12 and an internal reference. It is also specialized for the digitization of ECG signals since it can generate the right leg circuit, the Wilson central terminal, the Goldberger network and has several configurations to know the status of the electrodes. It should be noted that when it is in operation, it has low power consumption, and it can enter a hibernation state when it is not in operation to reduce power consumption [17].

The microSD card of the prototype developed does not have to be of a specific type, although 32 GB class 10 microSDHC memory of the ADATA brand was used for the performance tests. But, it is worth mentioning that the device only supports memories with a capacity of more than 2 GB up to 32 GB of the SDHC type and with a class higher than 4, but it is recommended to use class 10 to ensure a writing frequency in the microSD memory higher than 4 MB/s.

The ESP32 is designed for mobile applications, portable electronics and the Internet of Things, and it has all the most advanced characteristics of low-power chips, such as precise clock synchronization, multiple power modes and dynamic power scaling. The ESP32 contains a single- or dual-core 32-bit Xtensa LX6 Microprocessor capable of performing up to 600 million instructions per second and having a wide variety of peripherals highlighting 4 SPI modules, 18 ADC channels, 16 PWM channels, 10 touch sensors, 4 64-bit timers and Wi-Fi and Bluetooth communication. This device has a supply voltage of 2.3 V to 3.6 V and has a sleep mode that permits it to save power when not in use, which increases the battery life in portable devices [18,19].

The LCD display used in the design is the 1.44″ Colorful SPI TFT LCD display ST7735 128 × 128 [19], a liquid crystal display (LCD) variant that uses thin film transistor (TFT) technology to upgrade its quality. This display can be controlled via SPI communication protocol, has a supply voltage that can vary in the range of 3.3 V to 5 V and a white LED for backlighting that can be dimmed with a PWM channel. This display has 128 × 128 pixel resolution with a TFT driver (ST7735 [20]) that can display full 16-bit colors by using libraries available for Arduino and Raspberry Pi.

#### 2.2.1. CAECG Monitor User Interfaces

The user’s interaction with the device is developed through 5 buttons and the LCD screen. On the LCD screen, 8 views were implemented, each with different functions.

**Figure 5:**
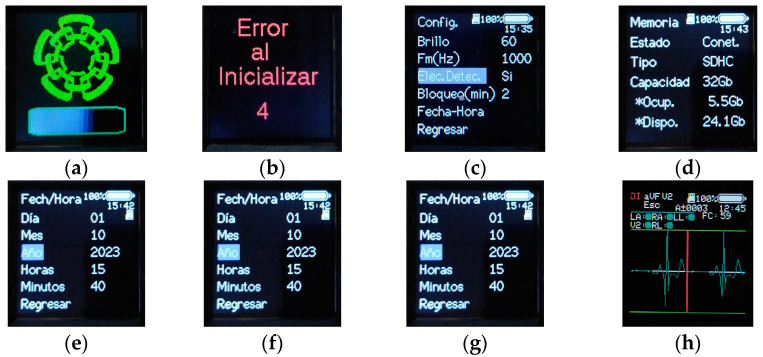
CAECG monitor views. (**a**) Start. (**b**) Error. (**c**) Main. (**d**) Memory. (**e**) Date–time. (**f**) Subject data. (**g**) Configuration. (**h**) Record.

Start: A view containing the CINVESTAV logo and a progress bar that advances as the ESP32 peripherals, the ADS1294 and the microSD memory are initialized. When the microSD memory is not inserted or there is a continuous error in the initialization and the attempts to initialize the memory have passed, the initialization of the rest of the peripherals is continued, and the status of the microSD memory is shown in the rest of the views (Figure 5a).

Error: Activated if an error occurs in the device operation or in the initialization of the ADS1294. This view performs a countdown, and after 10 s, the device is restarted; this cycle continues if the error persists (Figure 5b).

Main: It is displayed once the device has been initialized and no error occurred during initialization. From this view, it is possible to access the memory view, the configuration view and the patient data view by means of the new record view (Figure 5c).

Memory: It is possible to know, in more detail, the status of the microSD memory, being purely informative. In addition to knowing if it is connected or not, it is possible to know the total capacity of the memory, the occupied capacity, the available capacity and the type of memory (Figure 5d).

Date–Time: Allows one to modify the time and date of the system. More precisely, it allows one to modify the minutes, hour, day, month and year (Figure 5e).

Subject data: They are accessed from the new record option in the main view. In this view, the patient’s personal data can be established to differentiate them from the rest of the records, allowing for the definition of the subject’s name, age and sex. Once these parameters have been defined, the acquisition of the record is initiated through the Start Record option (Figure 5f).

Configuration: Its purpose is to modify the operating parameters of the monitor. It is possible to control the brightness of the device’s screen, which ranges from a maximum of 100 to a minimum of 0, with 60 being the default value. Define the sampling frequency of the device that can be 250 Hz, 500 Hz or 1000 Hz, which is the default frequency. The versatility of the device to select different sampling frequencies depends on the bandwidth used. The selection of the sampling frequency will depend on the physician or the researcher always taking into account the fact that a lower sampling frequency will have a lower consumption and the limit of the recording duration will be greater.

Enable the detection of the electrodes’ connection or not once the acquisition of the recording has started. Set the duration of time that the LCD screen remains on after a period of inactivity by using the Lock (min) option, which allows one to select between 1 min, 2 min, 3 min, 4 min, 5 min and permanently on. It should be mentioned that in all other cases, by excluding when a log acquisition is performed when only the LCD screen is turned off, the device goes into hibernation mode. In hibernation mode, the memory is disabled, the LCD screen is turned off and the ADS1294 and ESP32 are sent to sleep mode. To exit hibernation mode and reactivate the device, press any of the buttons until the device’s display turns on. The date–time option allows you to access the date–time view and modify the related parameters (Figure 5g).

Recording: In this view, it is possible to see the heart rate in real time with the HR parameter. To know the status of the electrodes by means of the labels RL, LL, LA, RA and V2, if the circle on the right is green, it means that the electrode has no connection problem, but if it is red, there are contact problems between the electrode and the subject’s skin. In addition, it is possible to select between the three leads DI, aVF and V2, which will be visible on the display. Finally, the HR parameter will show the heart rate of the selected lead since the heart rate is calculated independently for each lead with the algorithm developed in [11,21] (Figure 5h).

#### 2.2.2. Information Storage

**Figure 6:**
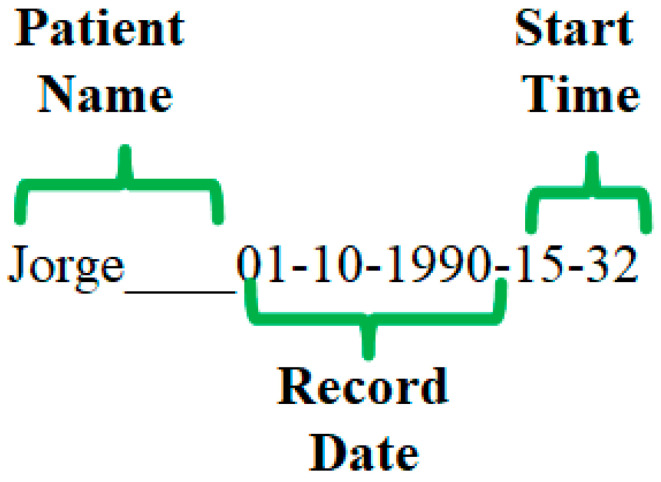
Format for the name of the directory where the records are stored.

To store the records obtained in the microSD memory, the device creates, in the root directory, a folder with the name of the subject, the date of the record and the start time of the record (Figure 6).

**Figure 7:**
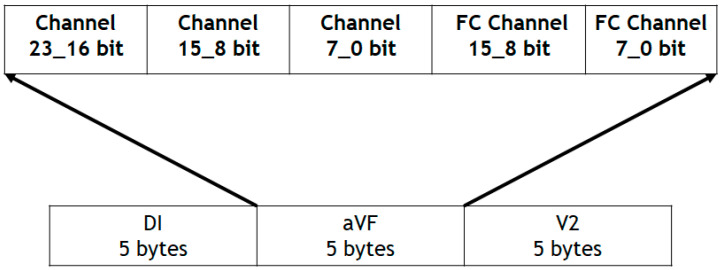
Data storage structure of the microSD memory.

Inside this folder are stored the files containing the information obtained from the ADS1294 and the QRS complex detection of each of the leads (DI, aVF and V2). The saved files are named Record0000, Record0001, …, Record9998 and Record9999. The Record0000 file stores the information collected in the subject’s data view, the sampling rate at which the ECG signals were acquired and the time that the CAECG monitor was on when the recording was started and ended. The rest of the files store the digitization results; as for the data obtained from the QRS complex detection, this information is stored with the structure shown in Figure 7.

Packets of 15 bytes are stored with 5 bytes per channel, where each channel is composed of the 24 bits obtained from the ADS1294 and 16 bits obtained from the QRS complex detection.

#### 2.2.3. CAECG Monitor Circuit Design

**Figure 8:**
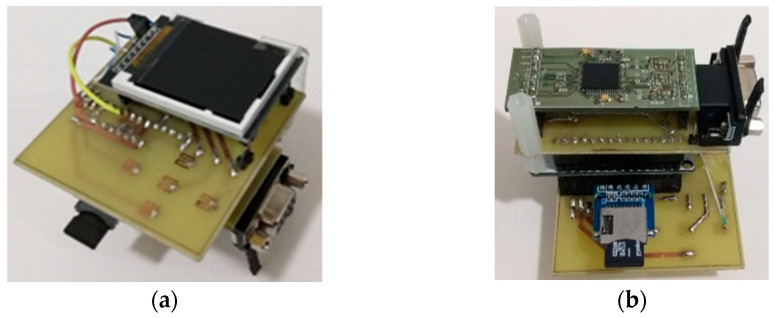
First prototype design of the CAECG monitor. (**a**) Top view. (**b**) Bottom view.

The CAECG monitor design was implemented in stages, which were inserted as boards with the ESP32 DEVKIT V1 development board as the nucleus (Figure 8).

**Figure 9:**
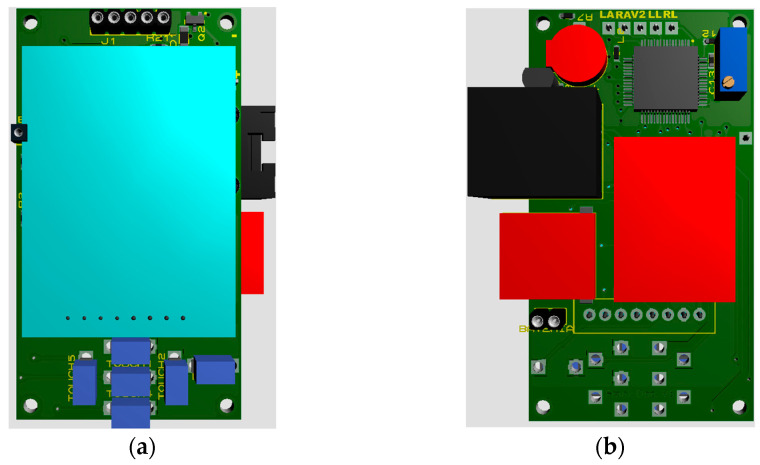
Printed circuit board. (**a**) Top side. (**b**) Bottom side.

In the first stage of development, the ADS1294 was added; in the second stage, the microSD memory was added; and in the third stage, the LCD screen and the touch buttons were added. When the operation of the prototype was validated, the process of reducing the size of the circuits began, first to two boards, one with the ADS1294 and the other with the rest of the components. The final version of the design was a board with approximate dimensions of 36.3 mm × 66.4 mm, as shown in Figure 9. This board has the buttons and the LCD screen on the upper side while the rest of the components are located on the lower one.

**Figure 10:**
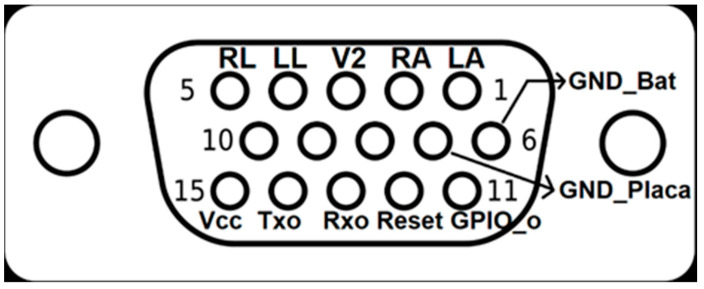
Pinning of the DB-15 connector.

A DB-15 connector was used to exchange the device with the outside. It is used for charging the battery, programming the device and exchanging information with the PC and the electrodes needed to acquire the ECG signal. The distribution of the pins on the DB-15 connector is shown in Figure 10.

In the DB-15 female connector connected to the CAECG monitor, the GND_Bat and GND_Placa pins are kept independent and the GND_Bat is connected directly to the battery GND, while GND_Placa is connected to the device after the battery protection circuit [22]. On the DB-15 male connector (connected to the communication and battery-charging module), the GND_Bat and GND_Placa pins are shorted; this procedure is performed to yield control over the battery to the charging circuit [16] and invalidate the battery-protection circuit. This procedure was necessary because both circuits conflicted when the battery voltage was lower than 2.5 V. The battery-protection circuit only restarts the current flow when a voltage higher than 3 V is present at the input. If the battery-charging circuit detects a voltage lower than 2.5 V, it enters a slow-charging state to recover the battery by sending only 6 mA until the voltage exceeds 2.5 V, whereby it enters fast-charging mode.

#### 2.2.4. CAECG Monitor Cabinet Design

**Figure 11:**
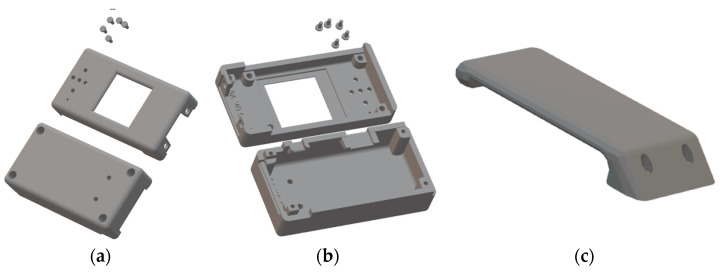
CAECG monitor cabinet design. (**a**) Top side. (**b**) Bottom side. (**c**) Support tab.

The Autodesk Inventor 2022 application was again used to design the cabinet. The box was designed to be printed with PLA, and we added metal grafts for the M2 screws (Figure 11a,b).

A demountable tab (Figure 11c) can be added to the designed cabinet, which is intended to facilitate the attachment of the prototype to clothing or a belt.

#### 2.2.5. Detection of the Electrodes’ Connection

To detect the electrodes’ connection, one of the functions of the ADS1294 is to inject a signal through the RL electrode and monitor the injected signal to ensure that it is present in each electrode during the signal digitization. For this purpose, the ADS1294 allows for the injection of several types of signals, and in this work, a 24 nA direct current was selected [17]. The report of the electrodes’ connection is obtained together with the data on the signal digitization in the ADS1294, which allows for the continuous monitoring of the electrodes’ status. This option is very important because it allows for the identification of a fault in the electrodes, either due to the deterioration of their performance in long-term recordings or due to an accident that causes their disconnection, avoiding the total loss of the recording.

### 2.3. Algorithm for Real-Time Heart Rate Detection

The algorithm for real-time heart rate detection is based on the algorithm presented in [11] using the CWT with splines reported in [13], which allows for its operation in real time with a low computational cost. This algorithm was adapted from the FPGA programming language in VHDL to the C programming language with the Arduino 1.8.2 software for implementation on the ESP32. To calculate the CWT, the steps described in ([11], Sections 2.1.2 to 2.1.5) were followed, but the coefficients used for the calculation were not multiplied by 216 since multiplication and division is possible in a microcontroller. In order to not repeat the description of the algorithm, only the changes made for the ESP32 implementation are mentioned below.

As mentioned in [21], the CWT realizes a bandpass filtering on the ECG signal that depends on the scale and the sampling frequency (Fm) selected in the setup, as shown in Table 1. The goal is to maintain a CWT bandwidth of 14.4 ± 0.1 Hz to 48.6 ± 0.2 Hz for any selected Fm to reduce the power line interference of 50 Hz and 60 Hz. Therefore, for the 3 Fm of 1000 Hz, 500 Hz and 250 Hz, the selected scales were 8, 4 and 2, respectively.

Once the CWT scale to be used has been defined, the algorithm starts by obtaining data from the ADS1294, and the CWT is performed on the data. It should be mentioned that although a CWT value is obtained for each piece of data obtained from the ADS1294, a shift or phase shift is introduced with respect to the ECG signal, which is approximately 48 samples for scale 8, 24 samples for scale 4 and 12 samples for scale 2. This results in a delay of 36 ms in the QRS complex detection in real time for the different sampling frequencies.

**Figure 12:**
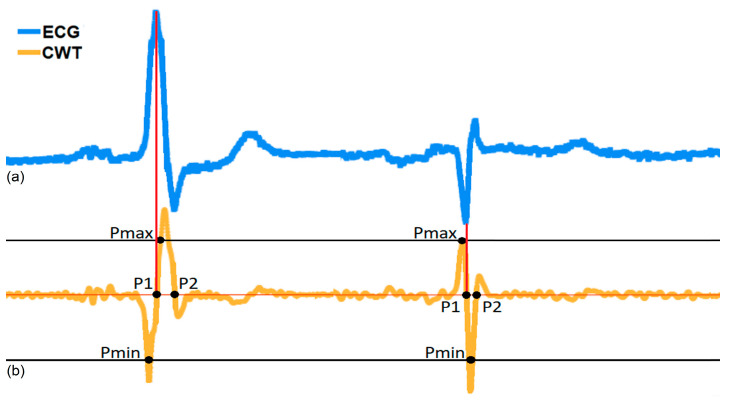
Example of the ECG signal with a sampling rate of 1000 Hz and the corresponding CWT on scale 2. (**a**) ECG signal; (**b**) CWT on scale 2.

**Figure 13:**
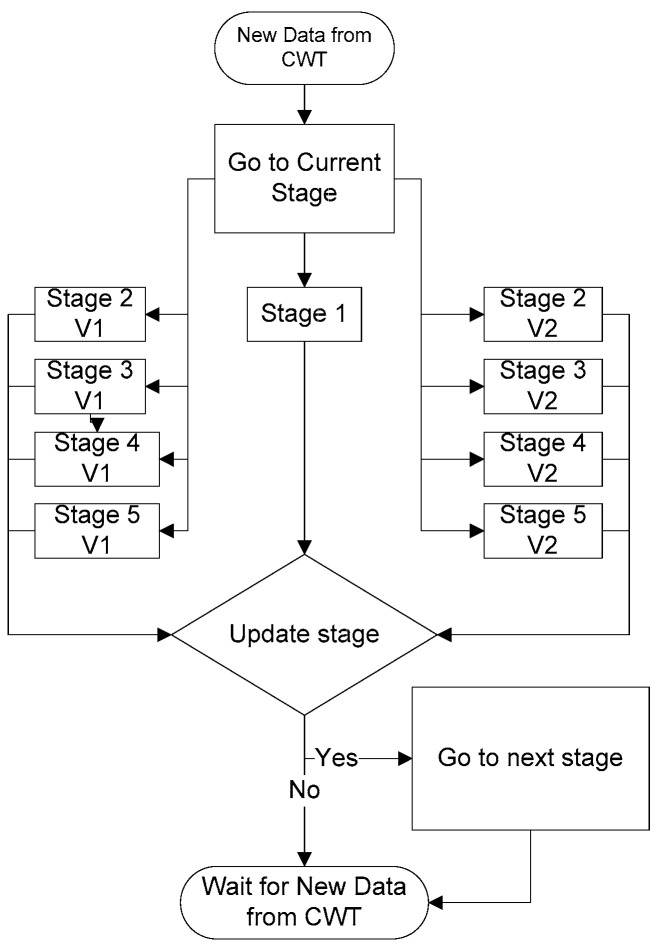
General algorithm for QRS complex detection in real time.

As the wavelet function used is the first derivate of a cubic B-spine of the 4th order expanded by two ([14], Figure 1), the QRS complex corresponds to a pair of moduli: a positive maximum (Pmax) and negative minimum (Pmin) that represent the falling slope and the rising slope, respectively, of the CWT in each different scale, as shown in Figure 12. To obtain the RR interval duration, the distance between the different zero crossings “P1” of two consecutive QRS complexes that correspond to the R or S waves peaks were measured. But, as shown in Figure 12, when there are QRS morphology changes, there are also changes in the order of the appearance of the CWT modulus, first the Pmin followed by the Pmax and vice versa. In this case, the QRS detection algorithm developed considers this change to obtain the P1 point. To identify this effect of the CWT on the QRS complex, two variables “Memory_Pmin” and “Memory_Pmax” were created, which have a value equal to 75% of the value obtained for the minimum and maximum peaks in the digitized ECG signal (Figure 13), respectively. The first stage is responsible for comparing whether the CWT result is less than “Memory_Pmin” or greater than “Memory_Pmax”, and then two different procedures can be followed, as described in ([11], Section 2.1.6).

### 2.4. CAECG Monitor Alarm System

**Figure 14:**
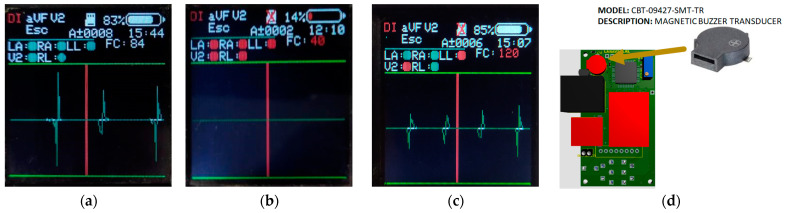
CAECG monitor alarm system: (**a**) Screen of correct system operation; (**b**) Screen of incorrect system operation; (**c**) Screen informing about some system problems; (**d**) Element in charge of the acoustic alarm.

The CAECG monitor has a real-time alarm system, and its purpose is to alert users about abnormal situations in its operation and the records acquisition. The alarms can be visual or acoustic. The visual alarms are shown on the LCD screen of the device (Figure 14), indicating the following situations:

- A fault in the electrodes’ connection (the electrode circle changes from green to red).
- The microSD memory disconnected (a red cross appears over the microSD memory symbol if it disconnected).
- The heart rate value is not between 60 and 100 beats per minute (the HR value changes from white to red).
- Low battery (the color of the battery bar changes from white to red)

The acoustic alarm uses the magnetic buzzer transducer (Figure 14d), which is activated with a PWM in the following situations:

- A fault in the electrodes’ connection.
- The microSD memory is disconnected during the ECG record acquisition.
- The microSD memory is full.
- The heart rate value is not between 60 and 100 beats per minute.

## 3. Results and Discussion

**Figure 15:**
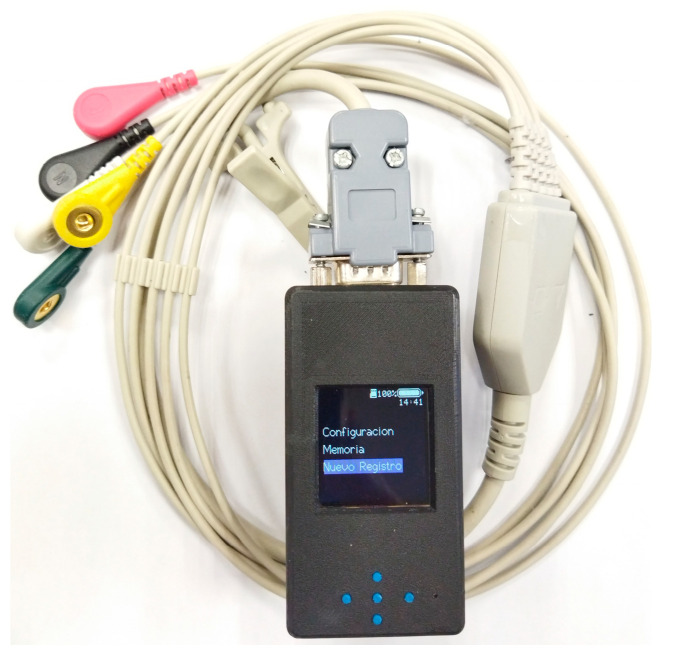
CAECG monitor developed with electrodes’ cable.

The CAECG monitor designed has two variants, one without the clamping tab with dimensions of 84.1 × 43.0 × 29.8 mm and a weight of 119 g. The second option is with the clamping tab with dimensions of 84.1 × 43.0 × 38.8 mm and a weight of approximately 129.1 g (Figure 15).

To validate the CAECG monitor, we reported the values of the common-mode rejection ratio (CMRR), bandwidth, full-scale range and resolution measured by the manufacturer [17] because no additional component was connected to the ADS1294. Also, we evaluated the ECG acquisition, the battery life and the minimum memory capacity required to store a 24 h ECG record by the monitor.

### 3.1. Evaluation of CAECG Monitor Parameters

The CMRR of the programmable gain amplifiers of the ADS1294 has a minimum of −105 dB at 50 Hz and 60 Hz. This high CMRR is required to reject noise and interferences in the ECG signal. The signal bandwidth is limited to half of the sampling frequency (Fm) according to the Nyquist sampling theorem. In this monitor, the options are 250 Hz, 500 Hz and 1000 Hz, and therefore the bandwidths will be 0 Hz to 125 Hz, 0 Hz to 250 Hz and 0 Hz to 500 Hz, respectively. For an ECG bandwidth of 150 Hz for adults and 250 Hz for infants, a minimal sampling frequency of 500 Hz is recommended [23], and for the analysis of high-frequency components in the ECG, 1000 Hz is used [5]. To modify the bandwidth, one can apply a digital filter.

The input differential dynamic range for the ADS1294 is determined by Equation (1) [17]:

(1) $$Full\_Scale\_Range=\frac{{\pm V}_{REF}}{Gain}=\frac{{2\times V}_{REF}}{Gain}$$

where V_REF_ for a 3 V supply is 2.4 V, and the gain can be programmed to have values of 1, 2, 3, 4, 6, 8 and 12. In this work, the ADS1294 was supplied with 3 V and the unity gain was maintained so the full-scale range is ±2.4 V.

The resolution of the digital analog converter is obtained by Equation (2), and the 24-bit resolution of the ADS1294 [17] was used:

(2) $${Resolution}_{adc}=\frac{V_{REF}}{2^{n}-1}=\frac{2\times 2.4\;V}{2^{24}-1}\approx 286.10\;nV$$

### 3.2. Battery Characteristics and Lifetime of the CAECG Monitor

The CAECG monitor uses a 103,665 battery of the Seamuing brand that is used for applications on the ESP32 development board. This battery has the following characteristics: Li-ion battery, no memory effect, voltage: 3.7 Vdc, current capacity: 3000 mAh, dimensions: 35 × 65 × 10 mm and approximate weight: 48.5 g.

The analysis of the maximum operating time of the prototype was performed at the maximum power consumption conditions, and this implies that electrode detection was activated, the LCD screen brightness was at maximum and the LCD screen was always on and at the highest sampling frequency (1000 Hz). These conditions represent a higher power consumption of the ADS1294, the microSD memory and the LCD display.

Under these conditions, the device consumption is 35 mA with maximum peaks of 50 mA; these peaks are due to the fact that the consumption for writing to the microSD memory is not constant and only occurs when the write buffers are full. But even so, for the theoretical calculation of the battery life, we use a constant consumption of 50 mA and a supply voltage of 3.3 V. With these defined values, we calculate the theoretical duration of the device operation.

Power consumed by the prototype:

P_d_ = 50 mA × 3.3 V = 165 mW

Average power delivered by the battery:

P_b_ = 3000 mAh × 3.7 V = 11,100 mWh

Theoretical battery life:

D_tb_ = P_b_/P_d_ = 11,100 mWh/165 mW = 67.27 h

The calculated battery life is possible if the battery is completely discharged. This situation does not occur in practice since the device stops working when the battery voltage is lower than 2.7 V because the battery protection circuit for low voltage enters into operation. Therefore, the battery retains between 30% and 40% of the charge; under these conditions, if the theoretical duration is reset to 70%, we obtain 47.09 h.

To validate the theoretical results, tests were performed with the device under the conditions previously established. First, the battery was charged until a voltage of 4.2 V (the maximum voltage), and then the acquisition of a record was started until the monitor was turned off and the procedure was repeated five times. With these conditions, an average battery life of 84 h was obtained; as expected, the value is higher than the theoretically calculated value because it was estimated with a higher operating current.

### 3.3. Minimum Storage Capacity for a 24 h ECG Record

Although the monitor developed can be used for short duration recordings, it is designed to obtain long-term continuous recordings (24 h or more), so the capacity of the microSD memory used must be taken into account. Each time the ADS1294 takes a sample, 15 bytes are stored (5 bytes for each channel, which are divided into 3 bytes for the channel sample and 2 bytes for the calculated heart rate), and with these conditions, the following equation was proposed:

(3) $${Storage}_{min}=15\left(\frac{bytes}{sample}\right)\times Fm\left(\frac{sample}{s}\right)\times t\left(s\right)$$

where Fm is the sampling frequency and t is the time in seconds. If we establish an Fm of 1000 Hz and a recording with a duration of 24 h, we obtain that a capacity of 1.21 GB is required for the recording:

$${Storage}_{min}=15\times 1000\times 86,400\approx 1.21\;\mathrm{GB}$$

Although the device can identify memories of 4 GB, 8 GB, 16 GB and 32 GB, it is recommended to use a memory of at least 8 GB in order to have approximately six records with a 24 h duration.

### 3.4. Validation of CAECG Monitor Acquisition

**Figure 16:**
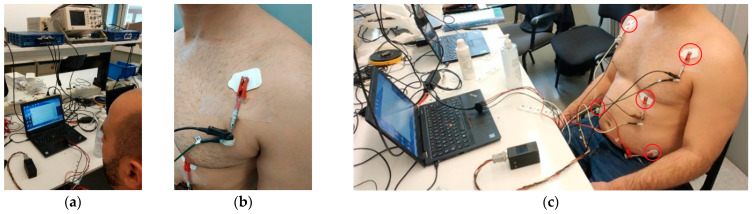
Simultaneous acquisition of the validation ECG record with the BIOPAC MP36 and the CAECG monitor: (**a**) BIOPAC MP36 and CAECG monitor connected. (**b**) Simultaneous connection of the electrodes. (**c**) Placement of 5 electrodes used for recording of leads DI, aVF and V2.

In order to verify that the CAECG monitor is capable of correctly digitizing the ECG signals, the results obtained by the monitor were compared with those obtained with the BIOPAC MP36 and its module for ECG LABEL SS2LB [24], which has the CE marking of the European Union for medical devices. To perform this validation, an ECG recording in the quasiorthogonal leads DI, aVF and V2 obtained from a subject was acquired simultaneously with the two devices at a frequency of 1000 Hz and a duration of 5 min (Figure 16a). In order to achieve simultaneous digitization, both devices were connected in parallel to each electrode, as shown in Figure 16b. To obtain the ECG recording in the three leads, five electrodes were connected in the positions shown in Figure 16c. The CAECG monitor, the BIOPAC MP36 and the computer used to store the information obtained from the BIOPAC MP36 used batteries to keep the subject isolated from the power supply line.

When the acquisition was completed, the first test was to analyze the morphologies of the signals, which are very similar, as can be seen in [12], Figure 7. To evaluate the visual statement, the correlation was calculated in the first 5 min after synchronizing the signals, obtaining a value of 91.78% [12]. When analyzing the records of the two devices, it was detected that the sampling frequency of the BIOPAC MP36 was 0.7 Hz higher than the CAECG monitor developed, a factor that explains why the correlation between the two digitized signals was not higher.

The second test to validate the quality of the digitized signal involved using the QRS complex to measure the RR interval in the two recordings to evaluate the agreement between them by using the statistical method of Bland–Altman [25]. For the R-wave peak detection in the ECG records, the algorithm presented in [14] was used. The results obtained show a high degree of similarity between the two RR detections shown in [12], Figure 8a. In the Bland–Altman plot shown in [12], Figure 8b, the mean error of the RR intervals from each recording was 1.00 ms, and the 95% confidence interval (±2 SD) was ±2.83 ms. This test showed that despite the difference of 8.2% between the ECG signals obtained by the CAECG monitor and the BIOPAC MP36, the location of the QRS complexes was not affected.

To evaluate the real-time detection of the QRS complexes of the CAECG monitor, the results were compared with those obtained with the algorithm [14] in Table 2. The sensitivity Se = TP/(TP + FN) and positive predictivity P+ = TP/(TP + FN) were calculated, where Tp is the number of positive detections, FN is the number of false positive misdetections and FN is the number of false negative detections. The error in the QRS detection was less than 5%, which allows for the real-time monitoring of the heart rate.

**Figure 17:**
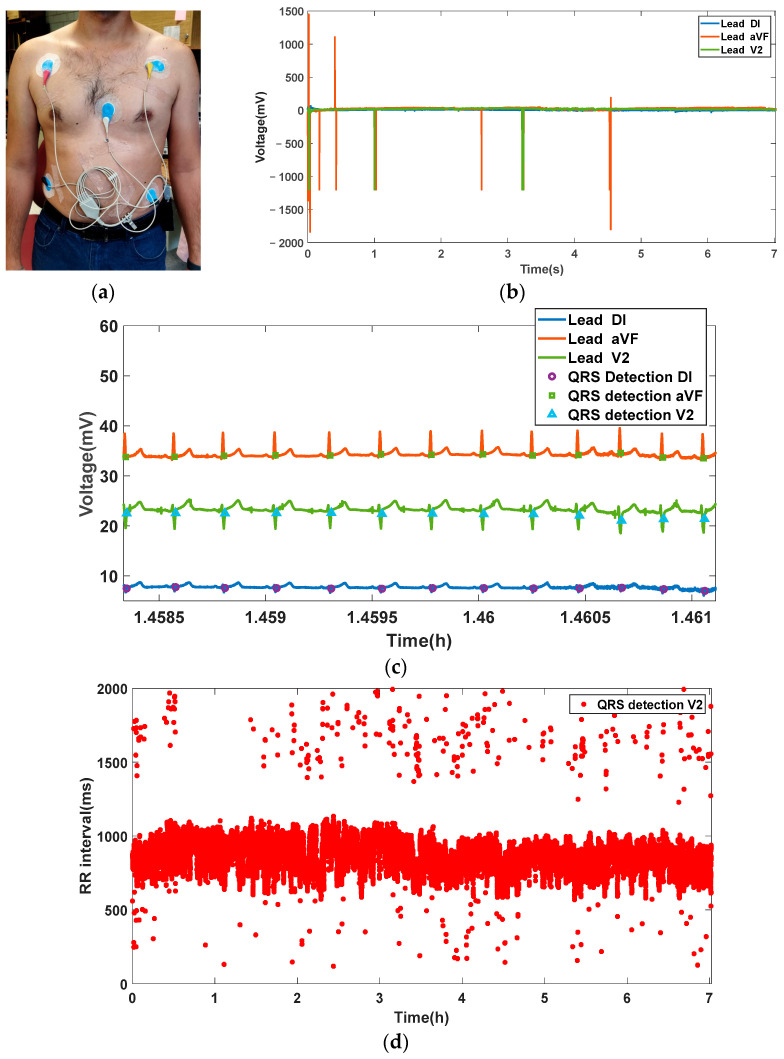
Acquisition of a 7 h ECG recording from a subject: (**a**) Test subject with CAECG monitor connected. (**b**) Complete recording of leads DI, aVF and V2. (**c**) Excerpt of the recording with detection of the QRS complex points in the 3 leads. (**d**) Beat-to-beat QRS complex detection in the V2 lead.

To finalize the validation of the CAECG monitor, an ECG record of three leads with the beat-to-beat real-time detection of the QRS complex from a subject was taken with a duration of approximately 7 h and is shown in Figure 17.

## 4. Conclusions

In this work, we present a low-power CAECG monitor for the simultaneous acquisition and storage of D1, aVF and V2 leads of long-term ECG recordings of an average approximate time of 84 h. Additionally, it allows for the continuous monitoring of the electrodes’ connection and a beat-to-beat heart rate measurement in real time of the 3 leads. It is also possible to obtain different leads modifying the electrodes position and to increase the number of ECG leads or the measurement of different biopotentials by replacing the analog-to-digital converter with an integrated ECG front end with small changes in the software and hardware. The use of the CWT with splines in the algorithm for real-time heart rate detection allows one to reduce noise and artifacts in the ECG signal.

Compared with a commercial monitor, the main advantages of the CAECG monitor are the heart rate measurement and storage in real time of the three leads and a higher sampling rate and resolution. The device is portable with dimensions of 84.1 × 43.0 × 29.8 mm and a weight of 119 g, but a tab can be added to facilitate its subjection to clothing or belts, which modifies the dimensions to 84.1 × 43.0 × 38.8 mm, and the weight increases by 10 g. The ECG acquisition of the CAECG monitor was validated with the commercial device BIOPAC MP36, obtaining a correlation between the digitized ECG records of 91.78%. This difference between the ECG records does not significantly affect the location of the R-wave peak due to the fact that, the agreement between the RR intervals measured in each recording calculated with the Bland–Altman method was ±2.83 ms.

The electrical characteristics of the CAECG monitor are a minimum CMRR of −105 dB because no electronic components were added to the ADS1924 input; a resolution of 286.10 nV; an input range of ±2.4 V; a current consumption of 50 mA; and a bandwidth ranging from zero to half of the sampling frequency, which can take values of 250 Hz, 500 Hz and 1000 Hz. It should also be noted that the device supports microSDHC memories from 4 GB to 32 GB as long as it is class 4 or higher.

To implement embedded algorithms for advanced processing offline or in real time, the clock frequency of the CAECG monitor can be increased to 240 MHz at the cost of increasing power consumption, and the ESP32 works with an expandable program memory of up to 16 MB.

Based on the feedback from the test subjects, there are plans to improve the design of the CAECG monitor cabinet and the way it fits the body to make it more ergonomic. This is due to the comments received during the tests, where they expressed a certain grade of discomfort due to the length of the cables and the fragility of the screen while they were sleeping or performing their daily tasks. In addition, they expressed that it is interesting to see the ECG in real time as well as to know their heart rate, the status of the electrodes and the microSD memory to request help or stop the recording acquisition if it is necessary. Also, they would like to see the three leads simultaneously.

The principal limitations of the device at present are the graphic interface, the power consumption and its dimensions. As future work, we plan to replace the LCD screen and buttons with a smartphone application by using the Bluetooth low-energy communication included in ESP32 in order to control the initialization parameters; the display of the signals; and the possibility of sending alarms in real time in the event of significant changes in heart rate, with the possibility of alerting a specialist remotely. This change has direct repercussions in the areas of energy consumption and the dimensions of the device since the LCD screen has a consumption of approximately 15 mA and eliminates the space occupied by the buttons.

The CAECG monitor will be used for the analysis of the dynamics of the HRV and HRT, which implies the detection of ectopic beats, and also for the development of our own ECG databases of short- and long-term ECG records of normal subjects and patients with cardiovascular and noncardiovascular diseases. These ECG databases will be used for the evaluation and testing of the performance of ECG processing algorithms. In addition, it is planned to implement an algorithm to detect the peak and end of the T wave to measure the RTpeak and RTend intervals and the dynamics of its dispersion in the three leads. Also, we are planning to implement an algorithm to detect ST segment changes that are related to cardiac ischemia.

## Figures and Tables

**Table 1:** Minimum (Fcmin) and maximum (Fcmax) cutoff frequencies obtained from the relationship between the sampling frequencies (Fm) and the CWT scales.

	Scale 2		Scale 4		Scale 8	
Fm	Fcmin	Fcmax	Fcmin	Fcmax	Fcmin	Fcmax
**250**	**14.3**	**48.3**	7.2	24.4	3.6	12.2
**500**	28.6	96.7	**14.4**	**48.7**	7.2	24.4
**1000**	57.3	193.4	28.9	97.5	**14.5**	**48.8**

**Table 2:** Comparison of QRS complex detection algorithms.

Record	Annotations	TP	FP	FN	Se %	P+ %
**Algorithm** [14]	313	313	0	0	100	100
**DI**	313	307	0	6	98.12	100
**aVF**	313	304	0	9	97.2	100
**V2**	313	313	0	0	100	100

## Data Availability

https://github.com/Frankms208/Long-term-continuous-ambulatory-ECG-monitor-of-3-simultaneous-leads.git (accessed on 28 August 2023).

## References

[1] World Health Organization (WHO) (2021). Cardiovascular Diseases (CVDs). https://www.who.int/news-room/fact-sheets/detail/cardiovascular-diseases-(cvds).

[2] Kennedy H.L., Zipes D.P., Jalife J. (2004). Use of long-term (Holter) electrocardiographic recordings. Cardiac Electrophysiology From Cell to Bedside.

[3] Goldberger J.J., Cain M.E., Hohnloser S.H., Kadish A.H., Knight B.P., Lauer M.S., Maron B.J., Page R.L., Passman R.S., Siscovick D. (2008). American Heart Association/American College of Cardiology Foundation/Heart Rhythm Society scientific statement on noninvasive risk stratification techniques for identifying patients at risk for sudden cardiac death: A scientific statement from the American Heart Association Council on Clinical Cardiology Committee on Electrocardiography and Arrhythmias and Council on Epidemiology and Prevention. Circulation.

[4] Steinberg J.S., Varma N., Cygankiewicz I., Aziz P., Balsam P., Baranchuk A., Cantillon D.J., Dilaveris P., Dubner S.J., El-Sherif N. (2017). ISHNE-HRS expert consensus statement on ambulatory ECG and external cardiac monitoring/telemetry. Ann. Noninvasive Electrocardiol..

[5] Crawford M.H., Bernstein S.J., Deedwania P.C., DiMarco J.P., Ferrick K.J., Garson A., A Green L., Greene H., Silka M.J., Stone P.H. (1999). ACC/AHA guidelines for ambulatory electrocardiography: A Report of the American College of Cardiology/American Heart Association Task Force on Practice Guidelines. J. Am. Coll. Cardiol..

[6] Health Quality Ontario (2017). Long-term continuous ambulatory ECG monitors and external cardiac loop recorders for cardiac arrhythmia: A health technology assessment. Ont. Health Technol. Assess. Ser..

[7] Enseleit F., Duru F. (2006). Long-term continuous external electrocardiographic recording: A review. Europace.

[8] Kennedy H.L. (2013). The Evolution of Ambulatory ECG Monitoring. Prog. Cardiovasc. Dis..

[9] Spacelabs Healthcare (2018). Lifecard CF. https://tinyurl.com/y6ae5ss6.

[10] Martínez-Suárez F., Alvarado-Serrano C. Prototype of an Ambulatory ECG Monitoring System with R Wave Detection in Real Time Based on FPGA. Proceedings of the 2019 16th International Conference on Electrical Engineering, Computing Science and Automatic Control (CCE).

[11] García-Limón J.A., Martínez-Suárez F., Alvarado-Serrano C. (2023). Implementation of Wavelet-Transform-Based Algorithms in an FPGA for Heart Rate and RT Interval Automatic Measurements in Real Time: Application in a Long-Term Ambulatory Electrocardiogram Monitor. Micromachines.

[12] Martínez-Suárez F., García-Limón J.A., Rivera-Cordova D., Flores-Nuñez L.I., Casas O., Alvarado-Serrano C. Long-Term Continuous Ambulatory ECG Monitor with Beat-to-Beat Heart Rate Measurement in Real Time using ESP32. Proceedings of the 2022 19th International Conference on Electrical Engineering, Computing Science and Automatic Control (CCE).

[13] Unser M., Aldroubi A., Schiff S. (1994). Fast implementation of the continuous wavelet transform with integer scales. IEEE Trans. Signal Process..

[14] Alvarado C., Arregui J., Ramos J., Pallàs-Areny R. Automatic detection of ECG ventricular activity waves using continuous spline wavelet transform. Proceedings of the 2005 2nd International Conference on Electrical and Electronics Engineering.

[15] (2005). Data Sheet.

[16] (2021). Datasheet.

[17] Texas Instruments (2012). Data Sheet ADS1294, ADS1294R, ADS1296, ADS1296R, ADS1298, ADS1298R. http://scholar.google.com/scholar?hl=en&btnG=Search&q=intitle:Low-Power+,+8-Channel+,+24-Bit+Analog+Front-End+for+Biopotential+Measurements#0.

[18] Systems E. (2019). ESP32 Series Datasheet. https://www.espressif.com/sites/default/files/documentation/esp32_datasheet_en.pdf.

[19] (2022). 1.44 Inch 128 × 128 Dots TFT LCD Display Datasheet.

[20] (2011). Controller ST7735S Datasheet.

[21] Martínez-Suárez F., Alvarado-Serrano C. VHDL module for the R wave detection in real time using continuous wavelet transform. Proceedings of the 2019 16th International Conference on Electrical Engineering, Computing Science and Automatic Control (CCE).

[22] (2016). DW01A Datasheet.

[23] Kligfield P., Gettes L.S., Bailey J.J., Childers R., Deal B.J., Hancock E.W., van Herpen G., Kors J.A., Macfarlane P., Mirvis D.M. (2007). Recommendations for the standardization and interpretation of the electrocardiogram: Part I: The electrocardiogram and its technology: A scientific statement from the American Heart Association Electrocardiography and Arrhythmias Committee, Council on Clinical Cardiology; the American College of Cardiology Foundation; and the Heart Rhythm Society: Endorsed by the International Society for Computerized Electrocardiology. Circulation.

[24] (2020). Hardware Guide Biopac MP36.

[25] Martin Bland J., Altman D.G. (1986). Statistical methods for assessing agreement between two methods of clinical measurement. Lancet.

